# Cell-type-specific alkaloid and terpenoid biosynthesis in glandular trichomes: single-cell and spatial transcriptomic perspectives

**DOI:** 10.3389/fpls.2026.1911787

**Published:** 2026-09-03

**Authors:** Hao Wei, Hengyu Zhang, Jinrong Wang, Jingyi Wei, Mengxin Zhu, Abinaya Manivannan

**Affiliations:** 1 School of Life Sciences, Qufu Normal University, Qufu, Shandong, China; 2 Shandong Key Laboratory of Wetland Ecology and Biodiversity Conservation in the Lower Yellow River, Qufu, Shandong, China; 3 Division of Life Science Department, Gyeongsang National University, Jinju, Republic of Korea; 4 School of Computational and Integrative Sciences, Jawaharlal Nehru University, New Delhi, India

**Keywords:** intercellular transport, metabolic compartmentalization, secondary metabolism, spatial omics, synthetic biology

## Abstract

Glandular trichomes serve as critical cellular factories for plant secondary metabolism, playing a central role in the biosynthesis, transport, and storage of diverse secondary metabolites. However, traditional macro-scale omics approaches often overlook the cellular heterogeneity within tissues, making it difficult to resolve the specific dynamics of different cell types in glandular trichomes. This limitation has hindered a deeper understanding of secondary metabolic pathways and their regulatory mechanisms. Recent advances in single-cell and spatial omics technologies are now helping to address this challenge. Single-cell omics enables researchers to elucidate the complex mechanisms of metabolic biosynthesis and intercellular transport at higher resolution. Meanwhile, spatial omics reveals their precise three-dimensional organization within intact glandular trichomes. By integrating these complementary approaches, researchers can identify candidate genes, spatial distribution of metabolic enzymes and transporters across distinct cell types, thereby constructing more refined and predictive metabolic network models. This review systematically summarizes the latest applications of single-cell and spatial omics technologies in research on glandular trichomes. Particularly focusing on the biosynthetic pathways of alkaloids and terpenoids in key model plants, including *Catharanthus roseus* (L.) G. Don, *Nicotiana tabacum* L., *Cannabis sativa* L., *Artemisia argyi* and *Artemisia annua*. It also envisions the promising prospects of deeply integrating single-cell omics, spatial omics, and synthetic biology. This integration will enhance the efficient bio-manufacturing of plant natural products and provide new technological avenues for sustainable development in agriculture and medicine.

## 1 Introduction

Trichomes are unicellular or multicellular appendages distributed on the surface of leaves, petals, stems, petioles, peduncles, and seed coats. They are regarded as specialized extensions of aerial epidermal cells (Johnson, 1975). The layer of trichomes covering the entire surface of an organ is termed an “indumentum”. Studies on the individual structures and collective characteristics of indumenta can be traced back to the early 17th century (Johnson, 1975). By the early 20th century, trichome research was primarily focused on morphology for plant classification (Metcalfe and Macqueen, 1951). The development of molecular biology approaches gradually shifted toward the initiation and developmental mechanisms of trichomes, as well as their functional roles in plant metabolism and defense systems (Levin, 1973; Champagne and Boutry, 2013; Markus Lange and Turner, 2013; Yang and Ye, 2013; Wang, 2014; Kaur and Kariyat, 2020).

Plants, being sessile organisms, are unable to escape environmental stresses and therefore evolved multiple adaptive strategies (Wang et al., 2021). One important strategy is the synthesis of bioactive “special metabolites,” also known as secondary metabolites, to enhance tolerance to both biotic and abiotic stresses (Huchelmann et al., 2017; Colinas and Alain, 2018). One of the key structures implementing this strategy is trichomes. It acts in synergy with epidermal stomata, cuticle, and the wax layer to contribute to plant defense and adaptation by synthesizing, storing, and secreting a variety of key compounds (McDowell et al., 2011; Hegebarth et al., 2016; Rakha et al., 2017).

Understanding the metabolic functions of trichomes requires access to pure and intact trichome cells. Over the past few decades, various single-cell isolation techniques to obtain trichome cells, including mechanical abrasion (Croteau and Winters, 1982), glass bead dissociation combined with density gradient centrifugation (Bergau et al., 2015), cryogenic grinding with dry ice powder (Yerger et al., 1992), microfluidic technology (Sims and Allbritton, 2007), fluorescence-activated cell sorting (FACS) (Gross et al., 2015), and laser capture microdissection (LCM) (Emmert-Buck et al., 1996; Espina et al., 2007) have been developed. These methods have played important roles in advancing research on trichome metabolite composition and gene expression (Xue et al., 2019). However, they have limitations such as sample fragmentation, limited yield, time-consuming procedures, cellular stress, and loss of spatial information (Xue et al., 2019; Livingston et al., 2020).

Beyond the inherent limitations of previous isolation techniques, a more fundamental challenge arises from the analytical approaches subsequently applied to the isolated cells. Traditional macro-scale multi-omics studies primarily rely on tissue-level bulk analysis, among which bulk RNA sequencing is a widely used classical technique. During the sequencing process, different cell types are mixed, and the resulting data reflect the average expression levels across multiple cell populations (Gross et al., 2015). This approach makes it difficult to resolve the functions of rare cell types and to determine whether specific signals originate from a particular cell type. This “signal dilution effect” is particularly pronounced in glandular trichome research, as they are usually limited in number and highly specialized in metabolism, making their cell-type-specific information easily masked by the background of whole tissues (Nakashima et al., 2016). Nevertheless, these classical methods have still laid an important foundation for elucidating the biosynthetic and transport mechanisms of secondary metabolites in plant glandular trichomes.

Recent advances in single-cell omics and spatial omics have introduced a new research paradigm that addresses these limitations (Tian et al., 2020; Rao et al., 2021). Single-cell RNA sequencing enables the resolution of cell type-specific gene expression within trichomes. Spatial omics further retains positional information, allowing reconstruction of the spatial organization of metabolic pathways, enzyme localization, and intercellular transport (Arulraj et al., 2024). The synergistic application of these technologies is fundamentally reshaping our understanding of trichomes as metabolic cell factories.

Building on this, the present study reviews recent findings to systematically evaluate the application and progress of single-cell and spatial omics in trichome metabolic research. It also examines how these approaches are reshaping our understanding of trichome secondary metabolism and its transport mechanisms. Further, the potential of multi-omics integration in elucidating glandular trichome function and advancing novel strategies for alkaloid and terpenoid biosynthesis has been discussed.

## 2 Trichomes as a reservoir of specialized metabolic cells

**Figure 1:**
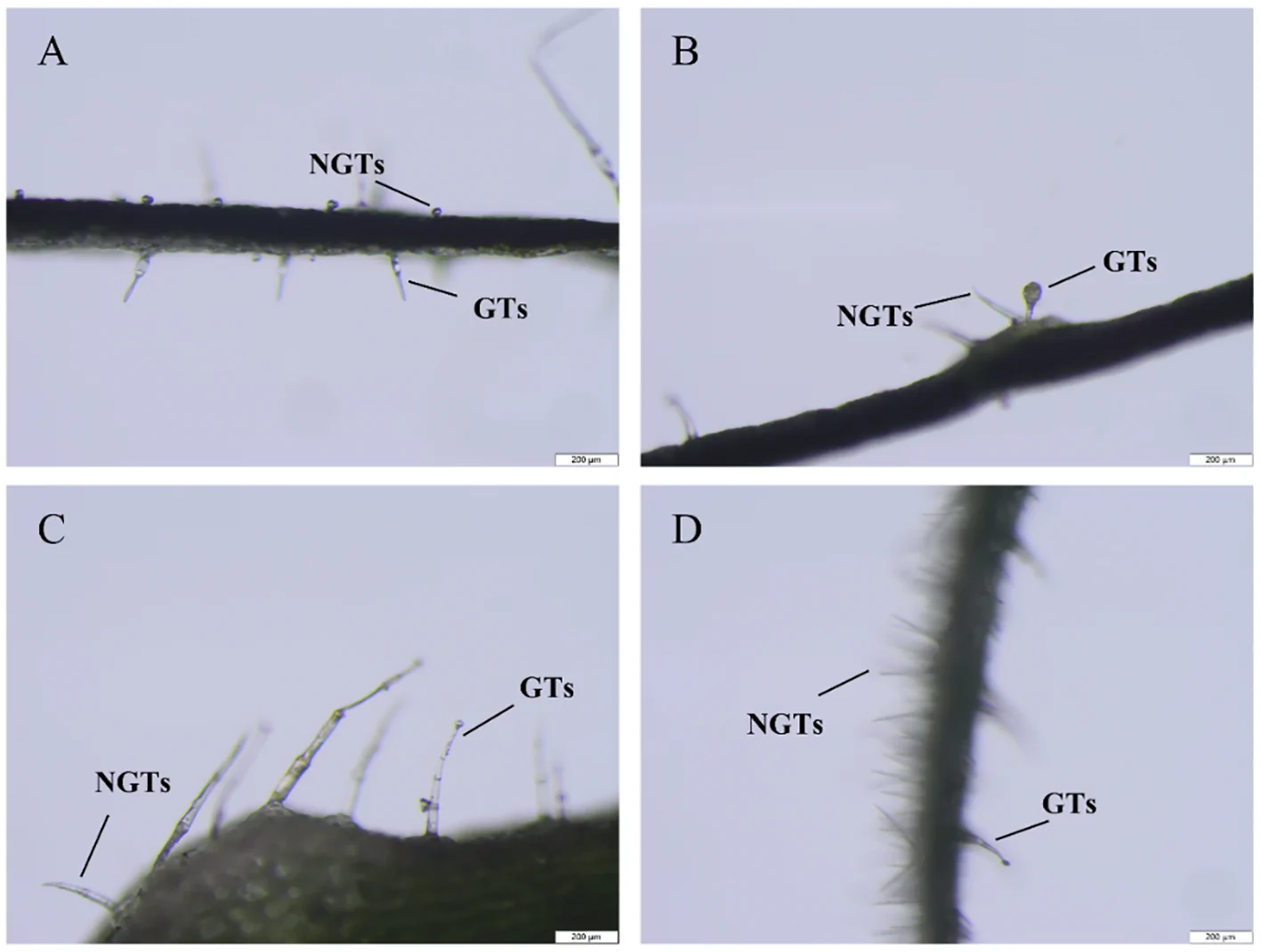
Microscopic observation of leaf trichomes in different plant species. **(A)** NGTs and GTs on the leaf epidermis of *Rehmannia glutinosa*; **(B)** NGTs and GTs on the leaf epidermis of *Lonicera maackii*; **(C)** NGTs and GTs on the leaf epidermis of *Primulina linearifolia* × *Primulina macrorhiza*; **(D)** NGTs and GTs on the leaf epidermis of *Broussonetia × kazinoki Siebold*. Scale bar = 200 *μ*m.

The term “trichome” is derived from the ancient Greek *trichos*, meaning “hair” (Schuurink and Tissier, 2020). These structures are distributed across the surface of aerial plant organs. Based on morphological and functional characteristics, trichomes are broadly classified into two types: non-glandular trichomes (NGTs) and glandular trichomes (GTs) (Werker, 2000; Wang et al., 2021) (**Figure 1**). NGTs are typically unicellular or consist of relatively simple multicellular structures (Koudounas et al., 2015). These are traditionally regarded as incapable of substantial secondary metabolite biosynthesis (Cao et al., 2020), however recent transcriptomic studies have detected the expression of secondary metabolism-related genes in NGTs (Stefanowicz et al., 2016). These findings suggest that NGTs may contribute to the broader metabolic network, but this topic is beyond the primary scope of the present review.

GTs are highly specialized and enriched in metabolic enzymes and transporters, and are widely recognized as the principal biosynthetic factories for plant secondary metabolites (Schuurink and Tissier, 2020).

GTs are diverse in morphological structures, encompassing peltate, capitate, and globular forms (Watts and Kariyat, 2021; Kaur et al., 2023; Hu et al., 2025). Their structural characteristics are closely related to their functions. A typical GT comprises of three distinct components such as base, stalk, and head (Kaur et al., 2023). The base is anchored to the epidermis and serves as the attachment point between the trichome and the leaf. It is generally composed of several square or disc-shaped basal cells that provide structural anchorage and nutritional support (Fuchs et al., 2022). The stalk consists of one or more cell files and commonly contains barrier cells with suberized lateral walls (Serrato-Valenti et al., 1997; Tissier, 2012). The head is composed of secretory cells capable of synthesizing and accumulating a broad spectrum of secondary metabolites, including terpenoids (Croteau and Winters, 1982; Gershenzon et al., 1992; Gershenzon and Dudareva, 2007), alkaloids (Olajire and Edewor-Kuponiyi, 2013), cannabinoids (Livingston et al., 2020), artemisinin (Zhao et al., 2024), phenylpropanoids (Gang et al., 2001; Deschamps et al., 2006; Xie et al., 2008), and flavonoids (Voirin et al., 1993; Tattini et al., 2000). In addition to these three core components, some studies propose that the epidermal cells surrounding the trichome base can be considered as part of its structure (Zhou et al., 2023). Despite their small size, GTs accumulate remarkably high concentrations of metabolites, underscoring their pivotal roles in plant defense and ecological adaptation. This phenomenon can be intimately linked to their specialized tissue architecture. Many metabolites are synthesized across distinct subcellular compartments and rely on primary metabolism for precursor supply (Allen et al., 2012). Whilst, high concentrations of metabolites can inhibit biosynthetic pathways or exert cytotoxic effects, plants employ strategies such as vacuolar sequestration, extracellular storage, and chemical modification to achieve detoxification and homeostatic maintenance (Aufschnaiter and Büttner, 2019; Yasseen and Al-Thani, 2022). In the secretory head of glandular trichomes, a subcuticular secretory cavity between the secretory cells and the cuticle, serve as a dedicated compartment for the deposition and storage of terpenoids and phenylpropanoids (Kim and Mahlberg, 1991; Sirikantaramas et al., 2005; Barbosa et al., 2016). Analogous structures are found in resin ducts, oil glands, and other secretory trichomes, collectively representing effective strategies by which plants mitigate autotoxicity (Zulak and Bohlmann, 2010; Huchelmann et al., 2017; Mewalal et al., 2017).

Nevertheless, secondary metabolic pathways in GTs frequently exhibit pronounced spatial compartmentalization, wherein distinct biosynthetic steps occur in different cell types and the final products must undergo intercellular transport to reach specialized storage structures (Roze et al., 2011; Martínez-Esteso et al., 2015; Cárdenas-Conejo et al., 2023). For instance, the precursors of certain alkaloids are synthesized in ordinary epidermal cells, while the final products are transported to idioblasts or laticifers (Murata et al., 2008; Guedes et al., 2024). In the capitate glandular trichomes of mint and cannabis, terpenoid scaffold formation occurs predominantly in stalk cells, whereas subsequent modifications and accumulation are concentrated in the head cells (Turner et al., 2000; Livingston et al., 2020; Conneely et al., 2021). Such intercellular metabolite flux involves the recognition and mediation by specific transporters, transmembrane or cell wall-traversing transport mechanisms. The selective utilization of symplastic versus apoplastic pathways remains one of the most challenging and central questions in plant metabolism research.

The complexity of the intercellular system of secondary metabolites in glandular trichomes constrains the advancement of metabolic engineering. When reconstructing complete metabolic pathways in microorganisms or model plants (Vidya Muthulakshmi et al., 2023), it is essential to account for critical intercellular transport steps. Otherwise, pathway intermediates may accumulate in unintended cell types, reducing the biosynthetic efficiency of the target compounds. Such mislocalization can also impose toxic effects on the host, ultimately compromising the engineered system. Thus, elucidating secondary metabolites and intercellular transport mechanisms in glandular trichomes carries both significant theoretical importance and practical value. With the rapid development of single-cell and spatial omics technologies, the spatial distribution of metabolic enzymes, transporters, and biosynthetic intermediates at cell-type resolution can be investigated. These advances facilitate the understanding of secondary metabolite biosynthesis and intercellular transport mechanisms in glandular trichomes.

## 3 Tools of resolution: single-cell sequencing and spatial omics

Single-cell omics dissects complex tissues into individual cells and characterizes their molecular profiles on a cell-by-cell basis, thereby overcoming the limitations of conventional omics approaches that obscure cellular heterogeneity (Liu et al., 2025; Wang and Agapito, 2025). Complementarily, spatial omics preserves the *in situ* positional information of cells within tissues (Liu et al., 2025). These two approaches enable simultaneous resolution of transcriptomic, metabolic, and spatial dimensions at the single-cell level, providing powerful tools for deciphering the biosynthesis, transport, and storage of secondary metabolites in GTs.

Achieving high-throughput single-cell analysis first requires the efficient and minimally disruptive isolation of individual cells or nuclei from complex plant tissues (Walker and Parkhill, 2008). As discussed previously, various trichome isolation strategies have been established, with microfluidics emerging as the dominant strategy owing to its high-throughput capacity and automation (Sims and Allbritton, 2007). Given the extremely low nucleic acid content at the single-cell level, early approaches relied primarily on polymerase chain reaction (PCR) amplification, which suffered from amplification bias and uneven genome coverage. Subsequently developed methods, including isothermal amplification, *in vitro* transcription (IVT), Phi29 DNA polymerase-based amplification, and multiple annealing and looping-based amplification.

Multiple annealing and looping-based amplification cycles (MALBAC), substantially improved amplification fidelity and efficiency. These methods also reduced sequencing blind spots, and advanced the overall reliability of single-cell omics (Walker and Parkhill, 2008; Zong et al., 2012; Kaur et al., 2019; Zhang et al., 2023).

**Figure 2:**
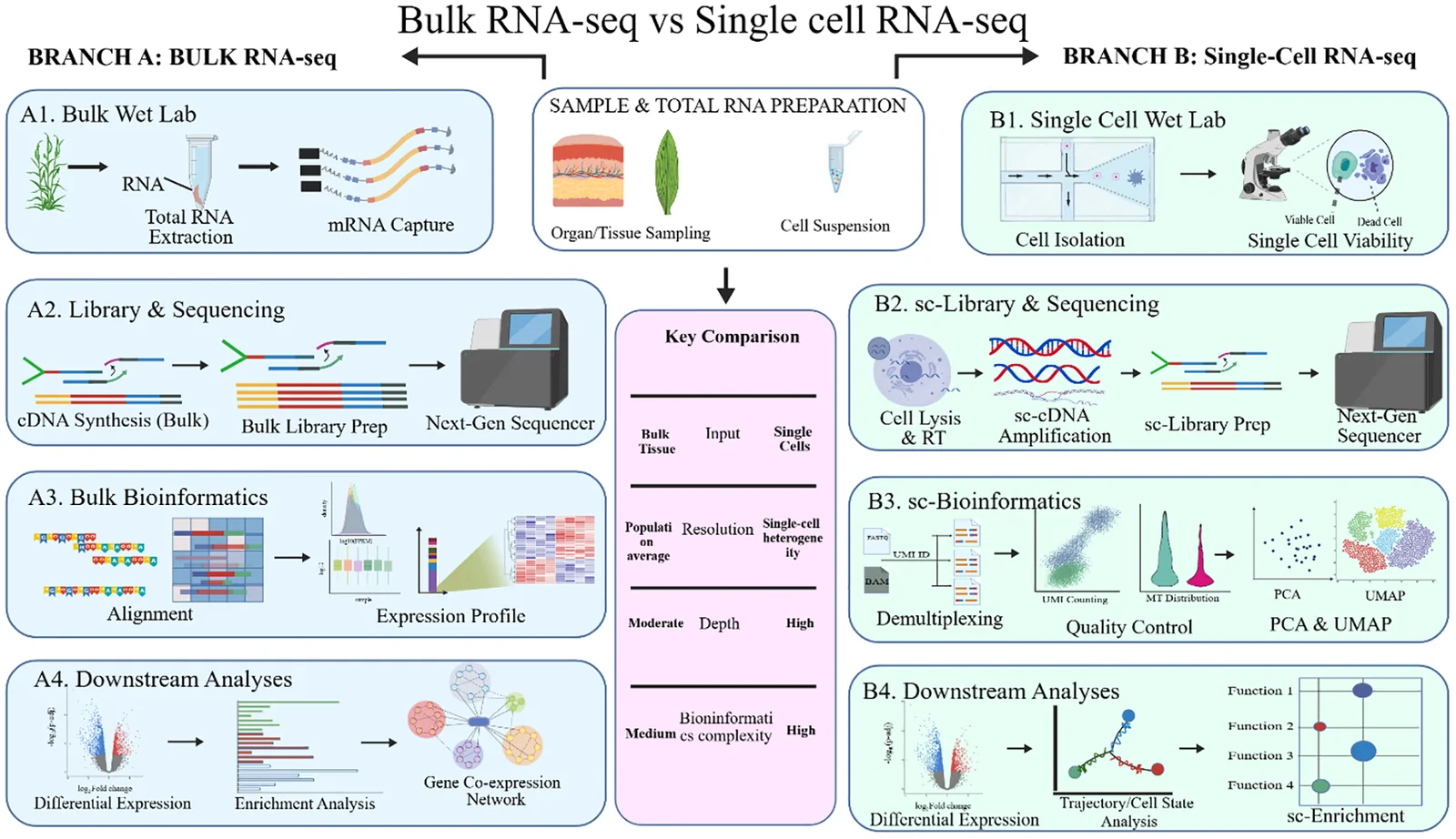
Comparison of bulk RNA-seq and single-cell RNA-seq workflows and key parameters. (modified from Ref (Tzec-Interián et al., 2025).). Both approaches share a common starting point of organ/tissue sampling and total RNA preparation. Branch A (Bulk RNA-seq): Total RNA is extracted from tissue followed by mRNA capture **(A1)**; cDNA synthesis, library preparation, and high-throughput sequencing are performed **(A2)**; bioinformatic processing includes sequence alignment and gene expression profiling **(A3)**; downstream analyses encompass differential expression analysis, enrichment analysis, and gene co-expression network construction **(A4)**. Branch B (Single-cell RNA-seq): Single-cell suspensions undergo cell isolation and viability assessment **(B1)**; following cell lysis, reverse transcription, single-cell cDNA amplification, and library preparation, sequencing is performed **(B2)**; the bioinformatic pipeline includes sample demultiplexing, UMI counting, mitochondrial gene proportion-based quality control, and PCA/UMAP dimensionality reduction **(B3)**; downstream analyses enable differential expression analysis, trajectory/cell state analysis, and single-cell enrichment analysis **(B4)**. Key differences between the two technologies are summarized in the central comparison table. This figure was created with BioGDP.com (Jiang et al., 2025).

Single-cell RNA sequencing (scRNA-seq) is the cornerstone of this field and has evolved rapidly since its introduction in 2009 (Tang et al., 2009). Compared with bulk RNA sequencing, which measures the average gene expression of a whole tissue, scRNA-seq resolves transcriptomic profiles at single-cell resolution (see **Figure 2** for a detailed workflow). Since then, a diverse array of protocols have been developed on distinct RNA capture and cDNA amplification strategies. Quartz-sequencing (Quartz-Seq) (Sasagawa et al., 2018) streamlines the original workflow and minimizes by-products. Cell Expression by Linear Amplification and Sequencing (CEL-Seq) replaces PCR with IVT (Hashimshony et al., 2012). And the Switching Mechanism At 5’ end of RNA Template-Sequencing (Smart-Seq) series, built on switching mechanism at 5’ end of RNA Template (SMART) technology, has been further developed into a commercialized platform (Ramsköld et al., 2012). These methods differ in their respective emphases on sensitivity, transcriptome coverage, and throughput. Protocol selection should be carefully weighed against sample type and research objectives.

With the advancement of scRNA-seq research and applications, simultaneously sequencing thousands to tens of thousands of cells in a single experiment has become increasingly necessary for characterizing transcriptional heterogeneity across cell populations. To meet this demand, microfluidic droplet barcoding technologies such as Droplet-based single-cell RNA sequencing (Drop-seq) and indexing droplets (In-Drop) were introduced in 2015. These approaches increased single-cell sequencing throughput and reduced cost greatly (Klein et al., 2015; Macosko et al., 2015). In these approaches, bead-based barcodes are co-encapsulated with individual cells within droplets, enabling each transcript to be traced back to its cell of origin and thereby facilitating scalable, traceable single-cell expression profiling.

Plant cells typically require enzymatic cell wall digestion for protoplast preparation, a process that induces stress responses and alters transcriptional profiles. Glandular trichome cells, being particularly fragile, are especially susceptible to damage during cell wall degradation (Mincarelli et al., 2018; Conde et al., 2021). Single-nucleus RNA sequencing (snRNA-seq) avoids this problem by isolating and sequencing nuclei directly, which substantially reduces transcriptional perturbations associated with cell dissociation (Chu et al., 2023). As a result, it is a better choice for studying fragile or hard-to-isolate cell types, such as glandular trichomes.

Currently, single-cell sequencing approaches rely predominantly on tissue dissociation, which inherently results in the loss of spatial positional information (Liao et al., 2023). Yet, spatial context is fundamental for understanding biological phenomena. For instance, during embryonic development, cells at different positions frequently adopt distinct fates (Kushner et al., 2014; Zou and Bai, 2019). In parallel with advances in single-cell technologies, spatial transcriptomics has emerged as another critical technology and has achieved substantial progress. Its defining advantage lies in the preservation of the original positional information of cells within their tissue context (Lubeck et al., 2014; Ståhl et al., 2016).

**Figure 3:**
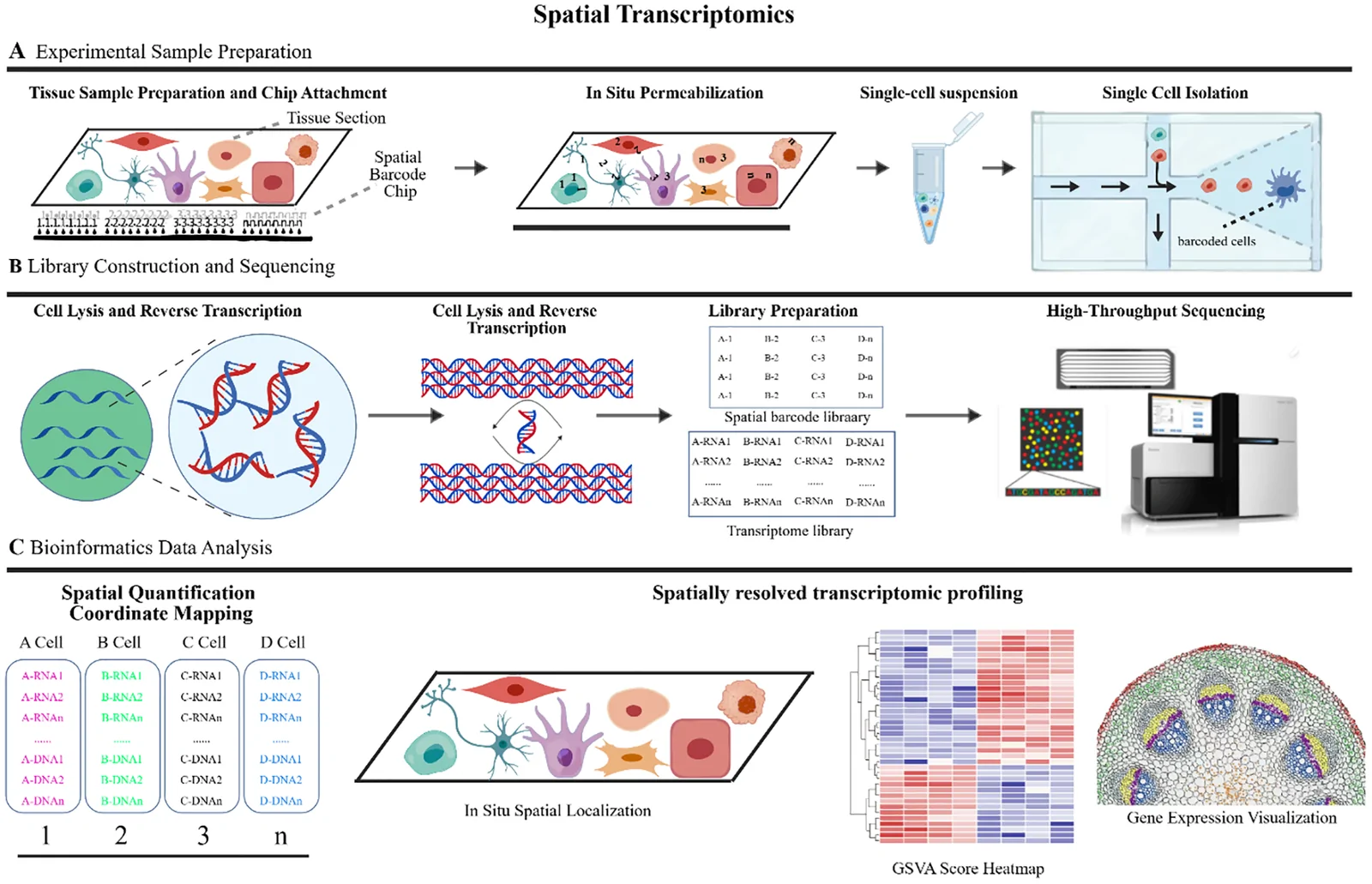
Schematic workflow of spatial transcriptomics technology. **(A)** Sample Preparation: Tissue sections are mounted onto a spatial barcoded chip; following permeabilization to release RNA, the material is processed into barcoded single-cell dissociated samples. **(B)** Library Construction and Sequencing: RNA is reverse-transcribed into cDNA, followed by library preparation and high-throughput sequencing to generate spatially barcoded transcriptomic data. **(C)** Bioinformatic Analysis: Sequencing reads are mapped to spatial coordinates, enabling *in situ* localization of gene expression, transcriptomic profiling (e.g., GSVA heatmaps), and tissue-level visualization. This figure was created with BioGDP.com (Jiang et al., 2025).

Spatial transcriptomics integrates high-throughput sequencing with tissue spatial information (see **Figure 3** for a detailed workflow) (Wang et al., 2025). Existing technologies fall into two broad categories. The first category achieves full-transcriptome coverage through spatial barcoding and sequencing, albeit with limited spatial resolution. The second category includes imaging-based approaches such as multiplexed fluorescence *in situ* hybridization (FISH) and *in situ* sequencing, that attain single-molecule or subcellular resolution but typically profile a more restricted set of genes (Ke et al., 2013; ChenKH, 2015; Shah et al., 2016; Ståhl et al., 2016; Wang et al., 2018; Rodriques et al., 2019; Liu et al., 2020; Alon et al., 2021; Rao et al., 2021; Zhuang, 2021; Chen et al., 2022; Moses and Pachter, 2022; Russell et al., 2024). Mapping single-cell clustering results onto spatial transcriptomic data, or using imaging-based methods to annotate cell types by their tissue position, provides complementary qualitative and quantitative spatial resolution. Recently, Wang Siyuan’s team developed a novel spatial transcriptomic method called Reverse-padlock Amplicon Encoding Fluorescence *In Situ* Hybridization (RAEFISH). This approach aims to overcome the conventional trade-off between transcriptome coverage and spatial resolution, offering a new avenue for *in situ* whole-genome single-molecule detection (Cheng et al., 2025).

The *in situ* distribution of metabolites is critical for understanding secondary metabolite biosynthesis and metabolic flux (Sun et al., 2023; Ogger and Murray, 2025). Spatial metabolomics — centered on mass spectrometry imaging (MSI) — enables direct mapping of metabolite spatial profiles across tissue sections. Commonly used ionization sources such as Matrix-assisted laser desorption/ionization mass spectrometry imaging (MALDI-MSI) (Caprioli et al., 1997; Sturtevant et al., 2016), desorption electrospray ionization mass spectrometry imaging (DESI-MSI) (Takáts et al., 2004; Song et al., 2023; Sun et al., 2023), laser ablation electrospray ionization mass spectrometry (LA-ESI-MS) (Nemes and Vertes, 2007; Meng et al., 2022), and secondary ion mass spectrometry (SIMS) (Hoppe, 2006; Rabbani et al., 2011). Emerging technologies such as Matrix-assisted laser desorption/ionization - secondary (MALDI-2) (Soltwisch et al., 2015), matrix-assisted laser desorption electrospray ionization (MALD-ESI) (Sampson et al., 2006; Nemes and Vertes, 2007), and laser ablation inductively coupled plasma (LA-ICP) (Sabine Becker, 2013; Meng et al., 2021)continue to expand detection capabilities. Although, MSI can simultaneously detect large numbers of known and unknown molecules, matrix effects, adduct formation, and in-source fragmentation complicate quantitative studies (Cordes, 2024). The introduction of isotopically labeled internal standards — such as ^13^C-labeled extracts — can improve quantitative accuracy (Zajaczkowski et al., 2025). However, the quantification and normalization of water-soluble small molecules remain principal challenges. Recently, Wang et al. (2025) developed an improved quantitative MSI workflow based on isotopically labeled ^13^C yeast extract as an internal standard to overcome these limitations, enabling the quantitative analysis of over 200 metabolic features (Wang et al., 2025).

Single-cell transcriptomics enables the resolution of transcriptional expression patterns at the level of individual cells, yet the rapidly growing data throughput places increasingly demanding computational requirements on single-cell transcriptomic data analysis (Lähnemann et al., 2020). Nevertheless, a relatively mature analytical pipeline has been established, encompassing key steps including raw data processing, quality control, normalization and batch correction, dimensionality reduction and clustering, trajectory inference, and cell type annotation (Wang et al., 2025).

During preprocessing, stringent filtering of low-quality cells which are characterized by abnormally low gene or unique molecular index (UMI) counts, or elevated mitochondrial/chloroplast gene proportions is required. — is required, along with the removal of doublets and ambient RNA contamination (Luecken and Theis, 2019; Fleming et al., 2023; Punzon-Jimenez et al., 2024). As plant cell walls must be removed by enzymatic digestion, which can introduce transcriptional perturbations (Mincarelli et al., 2018; Conde et al., 2021). As a result, snRNA-seq has emerged as an important alternative to mitigate enzymatic bias (Chu et al., 2023), although its comparatively lower transcript yield warrants particular attention. Post-Quality Control (Post-QC) data require normalization and batch effect correction to attenuate non-biological variation arising from differences in sample origin, tissue source, and enzymatic digestion (Chen et al., 2019; Zhang et al., 2019). Methods such as Seurat normalization (Gribov et al., 2010), DESeq2 (Love et al., 2014), correlation analysis (CCA)/recpirpocal PCA (RPCA) (Hao et al., 2024), Harmony (Korsunsky et al., 2019), Batch balanced k nearest neighbors (BBKNN) (Zhang et al., 2019), Liger (Liu et al., 2020), Combat (Johnson et al., 2007; Büttner et al., 2019)and Single-Cell Variational Inference (scVI) (Zheng et al., 2022) are widely employed, and are especially critical in plant single-cell studies characterized by tissue complexity and high material heterogeneity.

Corrected high-dimensional expression matrices are typically subjected to principal component analysis (PCA) for dimensionality reduction, followed by Uniform Manifold Approximation and Projection (UMAP) or t-Distributed Stochastic Neighbor embedding (t-SNE) for visualization of cell distributions (Maaten and Hinton, 2008; Becht et al., 2019; Chen et al., 2019; Huang et al., 2024). The quality of dimensionality reduction directly influences downstream clustering, as insufficient removal of low-quality cells can lead to the formation of spurious clusters (De Winther et al., 2023). Clustering commonly relies on community detection algorithms applied to graph-based representations, such as Louvain and Leiden (Blondel et al., 2008; Lambiotte et al., 2016; Traag et al., 2019), which have become the default approaches in frameworks including Seurat and Scanpy (Brendel et al., 2022; Guo et al., 2024); additionally, deep learning-based methods — such as the variational autoencoder-based PhytoCluster — have demonstrated superior accuracy and noise robustness on plant datasets (Wang et al., 2025).

To characterize cell developmental trajectories or state transitions, pseudotime analyses are frequently performed (Wang et al., 2024) using tools such as Monocle (Qiu et al., 2017), Slingshot (Street et al., 2018), and Partition-based graph abstraction (PAGA) (Wolf et al., 2019), inferring branching structures through graph fitting (Qiu et al., 2017; Rizvi et al., 2017; Chen et al., 2019), minimum spanning trees (Trapnell et al., 2014; Ji and Ji, 2016), or inter-cluster connectivity (Fletcher et al., 2017). In practice, comparing trajectory outputs from multiple algorithms is standard procedure to enhance the robustness of inference. Cell type annotation relies on marker genes (Chau et al., 2025); however, the limited availability of marker gene databases across plant species (Thibivilliers and Libault, 2021), gene multi-copy status (Chau et al., 2025), and functional divergence (Liu et al., 2021; Marand et al., 2021) render cross-species annotation particularly challenging. Beyond conventional manual annotation, automated tools such as SingleR (Aran et al., 2019), CHaracterization of cEll Types Aided by Hierarchical classification (CHETAH) (De Kanter et al., 2019), CellAssign (Zhang et al., 2019), and single-cell Cluster-based Automatic Annotation Toolkit for Cellular Heterogeneity (scCATCH) (Shao et al., 2020) are increasingly used. Cross-species integration methods based on orthologous gene anchors, including Seurat v3 (CCA and RPCA) (Stuart et al., 2019), Mutual Nearest Neighbors (MNN) (Haghverdi et al., 2018), Harmony (Korsunsky et al., 2019), and scVI (Lopez et al., 2018) are also becoming mainstream approaches. Beyond conventional manual annotation, automated tools such as SingleR (Aran et al., 2019), CHETAH (De Kanter et al., 2019), CellAssign (Zhang et al., 2019), and scCATCH (Shao et al., 2020)are widely utilized.

As plant single-cell sequencing frequently disrupts tissue spatial architecture, spatial validation through spatial transcriptomics or *in situ* hybridization is essential (Asgrimsdottir and Arenas, 2020), while computational spatial inference methods remain in their early stages of application in plant systems (Huizing et al., 2025). Overall, the core computational pathway from rigorous quality control and batch correction to effective clustering, trajectory inference, and annotation constitutes the foundation for translating single-cell data into biological interpretation. This enables researchers to identify cell types, resolve cell state transitions, and elucidate dynamic regulatory mechanisms underlying metabolic and developmental processes.

## 4 Case studies

Building on these technological advances, recent studies in plants with medicinal values have established important model systems for understanding the cellular organization of specialized metabolism. Although this review focuses on GTs, due to the limited availability of single-cell and spatial omics datasets, this section comprises both GT systems and well-characterized comparative models of spatially compartmentalized specialized metabolism. For instance, *Artemisia annua*, *Artemisia argyi*, and *Cannabis sativa* directly denote metabolism within GTs. However, *Catharanthus roseus* and *Nicotiana tabacum* are included due to the widely studied cell-type specialization, metabolic compartmentalization, and intercellular transport of secondary metabolites.

### 4.1 Artemisia argyi

The quality of *Artemisia argyi* Lévl. et Vant. (*A. argyi*) is closely associated with the development of glandular trichomes (GTs) (Cui et al., 2021; Luo et al., 2021). As specialized secretory structures, glandular trichomes serve as critical sites for the biosynthesis and storage of volatile terpenoids, particularly for the accumulation of diverse bioactive sesquiterpenoid compounds with pharmacological properties (Francis et al., 2004; Rasmann et al., 2005; Li et al., 2013). Previous studies have identified two types of glandular trichomes, GT-I and GT-II, on the leaf surface of *A. argyi*, which exhibit distinct differences in storage structures, cellular status, and maturation processes, indicating the complex cellular specialization underlying glandular trichome development (Cui et al., 2022). The rapid advancement of multi-omics technologies has greatly facilitated the elucidation of the biosynthetic mechanisms of characteristic secondary metabolites in *A. argyi*. Genome-wide analyses have revealed that the expansion of the *terpene synthase* (*TPS*) gene family may represent an important genetic basis contributing to the diversity of terpenoid compounds in *A. argyi (*Chen et al., 2023). Transcriptomic analyses have further identified multiple candidate genes involved in terpenoid biosynthesis and regulation, while functional characterization of *TPS* genes has provided insights into the molecular mechanisms underlying the formation of volatile compounds and mosquito-repellent activity in *A. argyi (*Yi et al., 2022; Zhang et al., 2022; Chen et al., 2023; Zhi et al., 2024). However, most previous studies have been conducted at the tissue or whole-organ level, limiting our understanding of the functional differentiation among distinct glandular trichome cell types during development and their specific contributions to the biosynthesis of bioactive compounds. Therefore, comprehensive investigation of the specialized developmental processes of glandular trichomes and their cell-type-specific metabolic regulatory networks is essential for elucidating the molecular basis of terpenoid biosynthesis in *A. argyi*.

Recently, Zhang et al. constructed the first high-resolution single-cell transcriptomic atlas of glandular trichomes in *A. argyi* and integrated it with metabolomic analyses to systematically reveal the cell-type-specific regulatory mechanisms underlying secondary metabolism in glandular trichomes (Dong et al., 2026). The study identified an EC_1 glandular trichome cell cluster and demonstrated that *lipid transfer protein* (*LTP*) and *peroxidase* (*POD*) genes were specifically and highly expressed in this cluster, contributing to the transport and secretion of secondary metabolites and serving as reliable molecular markers for glandular trichome identification (Sun et al., 2023; Sun et al., 2023). Furthermore, pseudo-time trajectory analysis identified 106 developmentally associated genes and 12 candidate transcription factors, revealing that members of the HD-ZIP, MYB, bHLH, and WRKY transcription factor families collectively regulate glandular trichome initiation, development, and maturation.

Furthermore, single-cell expression profiling revealed that genes associated with sesquiterpenoid biosynthesis were predominantly enriched in glandular trichome cells. By integrating co-expression analysis with enzyme activity validation, the study identified four glandular trichome-specific terpene synthase genes (*AarTPS52*, *AarTPS77*, *AarTPS95*, and *AarTPS96*). Among them, *AarTPS52* and AarTPS77 were demonstrated to encode β-farnesene synthase and β-caryophyllene synthase, respectively, and participate in the biosynthesis of key medicinal sesquiterpenoids. Promoter analysis further revealed that the regulatory regions of *TPS* genes were enriched with MYB/MYC binding motifs, suggesting that transcription factors may promote the glandular trichome-specific production of sesquiterpenoids through transcriptional regulation of TPS expression. Collectively, this study overcomes the limitations of conventional tissue-level investigations and, at the single-cell resolution, elucidates the intrinsic relationship among glandular trichome development, cellular differentiation, and sesquiterpenoid biosynthesis in *A. argyi*. It establishes a regulatory framework of “transcription factor–TPS–glandular trichome-derived metabolites”, highlighting that medicinal plant glandular trichomes are not merely passive storage structures for metabolites but rather dynamic metabolic units driven by cell-type-specific regulatory networks. However, the metabolic division of labor among different glandular trichome cell types, the mechanisms underlying precursor transport, and the regulation of terpenoid biosynthesis by environmental signals remain to be further elucidated.

### 4.2 Artemisia annua

Sweet wormwood (*Artemisia annua* L.) is an important model plant for studying GT-mediated secondary metabolism. The sesquiterpene lactone compound artemisinin produced by *A. annua* is a highly effective antimalarial drug (Liu et al., 2006; Lommen et al., 2006) and represents a classic example of a high-value plant-derived secondary metabolite. Artemisinin is primarily biosynthesized, secreted, and stored in glandular trichomes on the leaf surface, leading to the recognition of glandular trichomes as natural “metabolic factories.” Early studies based on glandular trichome observation, isolation, and histochemical analyses demonstrated that artemisinin predominantly accumulates in the subcuticular space of glandular trichomes. This indicated that these structures not only function in metabolite biosynthesis but also play essential roles in storage and compartmentalization (Olsson et al., 2009). However, due to the complex multicellular organization of GTs, conventional tissue-level approaches have been insufficient to resolve the specific contributions of distinct cell types to artemisinin biosynthesis.

Using laser microdissection pressure catapulting (LMPC) technology combined with gene expression profiling and immunolocalization analyses, researchers demonstrated that farnesyl diphosphate synthase (FPPS), the enzyme responsible for the biosynthesis of the artemisinin precursor FPP, is expressed across multiple cell types. In contrast, the key pathway enzymes amorpha-4,11-diene synthase (ADS), cytochrome P450 71AV1(CYP71AV1, and artemisinic aldehyde Δ^11(13)^ reductase (AAR) were predominantly localized in the apical secretory cells of GTs. These findings provided the first evidence of distinct spatial cellular specialization in artemisinin biosynthesis, in which certain cell types contribute to precursor supply, whereas secretory cells execute the core metabolic pathway (Olsson et al., 2009). However, this approach remains limited by its reliance on candidate gene detection and is insufficient for systematically resolving cellular heterogeneity and regulatory networks during glandular trichome development.

In recent years, single-nucleus RNA sequencing (snRNA-seq) and spatial transcriptomics technologies have provided new approaches for dissecting the cellular and spatial regulatory mechanisms underlying artemisinin biosynthesis. Recent studies have constructed a high-resolution cellular atlas of *A. annua* GTs, identifying 10 distinct cell types, including stalk cells, basal cells, and secretory cells, and revealed that glandular trichome development proceeds through initiation, intermediate differentiation, and terminal maturation stages. Single-cell analyses further demonstrated that artemisinin biosynthesis exhibits strict cell-type specificity. Key biosynthetic genes, including *ADS*, *CYP71AV1*, *ADH1*, *ALDH1*, and *DBR2*, are coordinately expressed primarily in secretory cells. Spatial transcriptomic analyses further validated their specific enrichment within GT regions, establishing a spatial regulatory model of **“**specialized secretory cells–specialized metabolic pathways–artemisinin accumulation**”** (Zhang et al., 2025). Furthermore, the identification of transcription factors such as *AaGSW1*, *AaGSW2*, *AaWRKY17*, and *AaMYB15* has provided new theoretical insights into the regulatory connections between glandular trichome development and artemisinin biosynthesis.

This case demonstrates that artemisinin biosynthesis is not a static metabolic process, but rather is jointly regulated by cellular developmental states, environmental signals, and genetic regulatory networks. For example, low-temperature stress can promote artemisinin accumulation by inducing jasmonic acid (JA) biosynthesis, which subsequently enhances the expression of ADS and DBR2. Meanwhile, coordinated regulation exists between the artemisinin biosynthetic pathway and other secondary metabolic pathways (He et al., 2024). Furthermore, artemisinin pathway-related gene expression and corresponding metabolites can still be detected in some GT-deficient materials, suggesting a certain degree of cellular plasticity in artemisinin biosynthesis. However, GT secretory cells represent the major and highly optimized production system under natural conditions. This research has advanced the understanding of plant GT-mediated secondary metabolism from tissue-level characterization toward single-cell spatial resolution, providing an important theoretical foundation for the precise regulation of high-value natural product biosynthesis (Judd et al., 2019).

### 4.3 Cannabis sativa

In Cannabis, GTs are responsible for the biosynthesis and storage of cannabinoids and a diverse array of terpenoids. Cannabinoids are predominantly synthesized and accumulated at high levels in the stalked GTs of female flowers, a finding well established through morphological observation and isolated trichome analysis (Braich et al., 2019). The trichomes possess an enlarged subcuticular storage cavity that serves as the primary spatial structure for cannabinoid enrichment (Gülck and Møller, 2020). Early studies revealed pronounced subcellular compartmentalization of cannabinoid biosynthesis: the MEP pathway in plastids supplies the monoterpene precursor Geranyl Pyrophosphate (GPP); the formation of olivetolic acid and Cannabigerolic acid (CBGA) is completed in the cytoplasm; and the terminal oxidative cyclization reactions are catalyzed by THCA synthase (THCAS) and CBDA synthase (CBDAS), which are secreted into the trichome storage cavity (Sirikantaramas et al., 2005). Paul et al., using hyperspectral coherent anti-Stokes Raman scattering (CARS) microscopy, further validated this spatial compartmentalization at the resolution of individual trichomes (Ebersbach et al., 2018).

Nevertheless, the molecular mechanisms underlying the intercellular transport of precursors such as GPP or CBGA across compartments remain unresolved. Recently (Wang et al., 2022), have identified multiple MATE and ABCG family members with high expression in floral tissues or trichomes, suggesting their potential involvement in transmembrane transport of cannabinoids or their intermediates (Wang et al., 2022). However, the substrate specificity of these candidate transporters and their precise localization within distinct cell layers or subcellular structures of the trichome have yet to be functionally validated. At the whole-tissue or trichome extract level, metabolomic studies have provided systematic analyses of cannabinoids and related metabolites, establishing an important foundation for elucidating chemotype variation across cultivars and metabolic regulation (Yeo et al., 2022; Stupak et al., 2025). More recently, studies integrating metabolomics with spatial localization strategies have achieved tissue-scale resolution of bioactive compound distributions within cannabis seed structures, revealing that the accumulation of cannabinoids and related metabolites exhibits distinct spatial organization (Li et al., 2026). Although such studies fall outside the primary focus of this review, these findings demonstrate that the incorporation of spatial omics approaches can substantially enhance our understanding of metabolite localization and transport routes, offering methodological insights and research directions for future application of spatial metabolic imaging, spatial transcriptomics, and single-cell multi-omics technologies within trichome systems.

### 4.4 Catharanthus roseus

*Catharanthus roseus* serves as the classical model plant for the biosynthesis of monoterpene indole alkaloids (MIAs). The MIAs consists of significant pharmacological value, with the anticancer compounds such as vinblastine and vincristine synthesized via a complex and highly compartmentalized pathway. Traditional studies employing RNA *in situ* hybridization and immunocytochemistry inferred that the biosynthesis of the core precursors vindoline and catharanthine involves intercellular transport of intermediates across distinct cell types (Yu and De Luca, 2014; Kulagina et al., 2022). However, such spatial inferences based on enzyme gene expression lacked direct evidence for metabolite localization at cellular resolution, limiting a complete understanding of the spatial logic underlying the biosynthetic pathway.

The development of single-cell omics technologies has provided critical breakthroughs in dissecting this multicellular compartmentalized pathway. For instance (Sun et al., 2023), employed scRNA-seq to localize 20 MIA pathway genes at the single-cell level for the first time (Sun et al., 2023). They showed that the Methyl-D-erythritol phosphate (MEP) pathway and iridoid precursor production occur mainly in internal phloem-associated parenchyma (IPAP) cells, whereas most downstream biosynthetic steps are enriched in epidermal cells. The terminal steps of vindoline biosynthesis were restricted to idioblasts, supporting a spatially staged transcriptional framework (Sun et al., 2023). At the metabolite level (Yamamoto et al., 2019), combined high-resolution imaging mass spectrometry with live single-cell mass spectrometry to achieve MIA metabolite localization at a spatial scale of approximately 10 μm (Yamamoto et al., 2019). Their results showed that most MIA precursors, such as iridoid intermediates, are enriched in epidermal cells. In contrast, vindoline, serpentine, and related compounds are markedly enriched in idioblasts and laticifers, thereby corroborating at the metabolite level the multicellular division of labor and intercellular transport requirements in MIA biosynthesis and accumulation (Yamamoto et al., 2019).

Building on this foundation (Kang et al., 2025), developed a single-cell multi-omics workflow enabling the simultaneous acquisition of scRNA-seq and single-cell mass spectrometry data from the same protoplast, achieving quantitative coupling of gene expression and metabolite abundance (Kang et al., 2025). Intermediates such as secologanin were found to co-localize strongly with epidermal cell markers, while terminal products including serpentine and anhydrovinblastine were concentrated in idioblast regions. Vindoline concentrations showed strong positive correlations with its terminal biosynthetic genes such as desacetoxyvindoline 4-hydroxylase (D4H), deacetylvindoline acetyltransferase (DAT), and N-myristoyltransferase (NMT), whereas upstream genes exhibited weaker correlations with end products, reflecting extensive intercellular transport of intermediates. This single-cell correlation analysis not only validated the spatial compartmentalization of the MIA pathway but also provided data-driven support for identifying putative transport nodes. For instance, the expression pattern of NRT1/PTR FAMILY 2.4 (NPF2.4) was found to closely parallel the accumulation of iridoid intermediates, suggesting its potential involvement in the import of precursors such as secologanin into epidermal cells (Kang et al., 2025). Earlier molecular biology studies confirmed that the epidermis-specific G-type ATP-binding cassette (ABCG) transporter catharanthine plasma membrane exporter (CrTPT2) mediates the efflux and secretion of catharanthine to the leaf surface (Yu and De Luca, 2013). These findings complement the spatial framework revealed by single-cell and spatial omics, thereby supporting a comprehensive understanding of the multicellular metabolic network underlying MIA biosynthesis.

Overall, studies on *C. roseus* provides key insights into MIA biosynthesis by integration of single-cell transcriptomics and spatial metabolomics to identify candidate transport nodes. Even though, this system is not based on GTs, it provides a valuable model for understanding spatial organization of specialized metabolism.

### 4.5 Nicotiana tabacum

The biosynthesis of nicotine in *Nicotiana tabacum* is a promising model of inter-organ metabolic specialization. In *N.tabacum* the biosynthesis, transport, and accumulation of nicotine are spatially separated between roots and aerial parts. Root is the major site of nicotine biosynthesis, with vital pathway enzymes expressed in root tissues. After biosynthesis the transport and accumulation of nicotine occurs in areal parts. Therefore, the inclusion of *N. tabacum* in this review highlight the significance of inter-organ transport of secondary metabolite. In detail, nicotine biosynthesis in tobacco exhibits a characteristic “organ-level division of labor”: key enzymes in the nicotine biosynthetic pathway, including quinolinate phosphoribosyltransferase (QPT), putrescine N-methyltransferase (PMT), and N-methylputrescine oxidase (MPO), have been systematically identified in root tissue through molecular cloning and expression analysis. Thus, establishing the root as the primary site of nicotine biosynthesis. Nicotine is subsequently transported via long-distance phloem transport to aerial tissues, where it accumulates in epidermal cells and glandular trichome structures (Zenkner et al., 2019).

At the leaf level, tobacco GT exhibit pronounced functional differentiation into two distinct types: long-stalked glandular trichomes (LGTs) and short-stalked glandular trichomes (SGTs). Early ultrastructural studies revealed that LGTs secrete resinous lipophilic substances, with head cells enriched in chloroplasts and electron-dense inclusions, whereas SGTs do not secrete resin but can exude nicotine-containing aqueous solutions under high-humidity conditions (Meyberg et al., 1991). This marked divergence in structure and secretory products suggests that distinct trichome types may fulfill different functional roles in metabolite biosynthesis, transport, and secretion. Bulk omics analyses of mixed leaf tissue are confounded by background signals from non-trichome cells, limiting precise spatial attribution of biosynthetic and transport mechanisms to specific trichome types (Yeo et al., 2022).

The introduction of single-cell technologies has substantially enhanced spatial resolution (Chen et al., 2024). constructed a single-nucleus transcriptomic atlas of tobacco leaves, successfully capturing and distinguishing LGT and SGT cell clusters (Chen et al., 2024). Key enzyme-encoding genes involved in diterpene biosynthesis, including terpene synthase (NtCPS2), *Nicotiana tabacum* ABS, and *Nicotiana tabacum* Cytochrome P450 71D16, were found to be enriched specifically in LGTs, while SGTs showed preferential expression of genes associated with cell differentiation and distinct secretory processes. These findings establish a molecular foundation for dissecting the metabolic division of labor within trichomes and the intercellular transport networks that underpin it (Chen et al., 2024).

In contrast to the functional differentiation of leaf glandular trichomes, the tissue-level localization of nicotine biosynthesis in roots has been established through molecular biology approaches. More recently, snRNA-seq has provided a cellular-resolution framework in support of this model (Jin et al., 2024) performed the first single-nucleus transcriptomic analysis of tobacco seedlings, identifying 29 cell clusters corresponding to 18 cell types (Jin et al., 2024). A subsequent report presented at the 2024 Cooperation Centre for Scientific Research Relative to Tobacco (CORESTA) conference further demonstrated that the majority of genes involved in nicotine biosynthesis are specifically expressed in root cortical cells, a finding validated by *in situ* hybridization and spatial metabolomics (Construction and utilization of tobacco cell landscape at single-cell level, 2024).

Within this inter-organ pathway, transporters constitute critical molecular bridges connecting each biosynthetic stage. The purine uptake permease (PUP) family member nicotine up take permease (NUP1) localizes to the root tip plasma membrane and is responsible for retrieving apoplastic nicotine back into the cell (Hildreth et al., 2011). The multidrug and toxic compound extrusion (MATE) transporter *Nicotiana tabacum* jasmonate-inducible alkaloid transporter 1 (Nt-JAT1) is co-upregulated with nicotine biosynthetic genes in response to methyl jasmonate (MeJA) induction and has been demonstrated to mediate transmembrane nicotine transport via a proton antiport mechanism (Morita et al., 2009). The leaf-specific jasmonate-inducible alkaloid transporter 2 (JAT2) localizes to the tonoplast and participates in vacuolar sequestration of nicotine within leaf cells (Shitan et al., 2014b; Shitan et al., 2015), while root-expressed Multidrug and toxic compound extrusion1/2 (MATE1/2) are involved in transient vacuolar storage of nicotine at the site of biosynthesis (Shitan et al., 2014a). Together, these components constitute a multilayered transport network encompassing biosynthesis, transient storage, long-distance transport, and terminal accumulation of nicotine.

Taken together, the case studies of representative trichome-bearing plants such as *Artemisia argyi, Artemisia annua*, and *Cannabis sativa* along with *Catharanthus roseus*, and *Nicotiana tabacum*, have employed single-cell and spatial omics technologies to illuminate the biosynthetic and transport mechanisms of specialized metabolites. To clearly present the distinctive research characteristics and key findings across species, the essential information from these studies are systematically summarized below (**Table 1**).

**Table 1:** Summary of metabolic mechanism studies in trichome-bearing plants based on single-cell/spatial omics.

Species	Metabolites	Methods	Spatial/cell-type validation	Metabolite localization evidence	Functional validation	Key transcription factors	Key transporters	References
*Artemisia annua*	Artemisinin	snRNA-seq; spatial transcriptomics; RNA *in situ* hybridization; qRT-PCR; LC-MS/MS	GST subtypes were resolved at single-nucleus resolution; GST domains and marker genes were validated by spatial transcriptomics and RNA *in situ* hybridization.	Indirect/tissue-level. Developmental LC-MS/MS quantified artemisinin and precursors; cell-level metabolite imaging was not reported.	Expression and marker validation were performed; newly identified hub genes remain candidates pending genetic validation.	AaGSW1/2, AaMIXTA, AaTAR1, AaTLR3, AaWIN1, AabHLH1, AaMYC2, AaWRKY9/17,	PDR1; LTP-related candidates	(Zhang et al., 2025)
*Artemisia argyi*	Sesquiterpenoids	scRNA-seq; LC-MS/GC-MS; RT-qPCR; co-expression analysis; *in vivo*/*in vitro* TPS assays	The EC_1/GT cluster was identified by single-cell sub-clustering, and GT marker enrichment was validated in isolated GTs by RT-qPCR.	Trichome-type level. GT-vs-NGT metabolomics showed GT enrichment of sesquiterpenoids and other secondary metabolites; cell-level MSI was not reported.	AarTPS52, AarTPS77, AarTPS95 and AarTPS96 were biochemically characterized	AarTPS52, AarTPS77, AarTPS95, AarTPS96;	LTP genes (candidate lipophilic metabolite export/cuticle-secretion genes)	(Dong et al., 2026)
*Andrographis paniculata*	Diterpene lactones	DESI-MSI scRNA-seq	Single-cell clusters were integrated with tissue-level spatial metabolite maps.	DESI-MSI provided spatial localization of major diterpene lactones.	ApHY5 and ApPIF1 were biochemically validated by dual-luciferase reporter assays to activate ApCPS2 promoter; light-induction experiments further supported the regulatory role.	ApHY5, ApPIF1	/	(Zeng et al., 2026)
*Camptotheca acuminata*	Camptothecin	scRNA-seq	Cell-type transcript evidence was provided; independent tissue/cell spatial validation was not reported.	NR^*^	Functionally validated heterologously: CaMYB5 and CaTT8 (co-expressed with CrIDM1) activated MIA biosynthetic genes in *C. roseus* petal transient overexpression assays.	CaMYB123, CaMYB5, CaTT8, CaBIS	/	(Li et al., 2026)
*Catharanthus roseus*	MIAs	scRNA-seq	Cell-type transcript evidence was provided; independent tissue/cell spatial validation was not reported.	NR	Transient overexpression of CrIDM1, CrIDB1, CrIDM4, and CrBIS1 in *C. roseus* petals activated MIA biosynthetic genes; promoter transactivation assays confirmed feedback regulation of CrIDB1 by CrIDM1+CrIDB1.	CrIDM1, CrIDM4, CrIDB1, CrBIS1	/	(Li et al., 2026)
*Catharanthus roseus*	MIAs, flavonoids	scRNA-seq	Matched transcript-metabolite profiles were generated from individual cells/protoplasts.	Single-cell MS supported metabolite localization and transcript-metabolite correlation.	Metabolite-gene correlations were validated at single-cell level; transporter functions are candidate or supported by previous studies.	D4H, DAT, NMT	NPF2.4, TPT5	(Kang et al., 2025)
*Catharanthus roseus*	MIAs	scRNA-seq, Hi-C	Cell-type transcript and chromatin-contact evidence were provided; independent tissue spatial validation was not reported.	NR	VIGS of SLTr transporter confirmed its role in secologanin transport; heterologous expression of THAS1/THAS2 validated the *in vitro* reductase activity toward iminium dimer to form AHVB; VIGS of THAS1/THAS2 also performed.	ORCA4, THAS1, THAS2	MATE family transporter SLTr	(Li et al., 2023)
*Catharanthus roseus*	MIAs	scMS Imaging MS	Cell/tissue metabolite mapping was performed, although not by transcriptomic spatial omics.	Imaging and live single-cell MS localized MIAs to epidermal cells, idioblasts and laticifers.	Direct metabolite localization was provided; genetic functional validation was not reported.	T16H2	NPF family transporters	(Yamamoto et al., 2019)
*Nicotiana tabacum*	Nicotine	snRNA-seq	snRNA-seq distinguished trichome/cell clusters; root localization of nicotine biosynthesis is supported by related *in situ* and spatial metabolomics evidence.	Organ/tissue level for nicotine localization; direct cell-level metabolite imaging in the cited snRNA-seq atlas was not reported.	Transporter activities for NUP1, JAT1/2 and MATE1/2 were validated in prior molecular studies; snRNA-seq provides cell-type context.	/	LTPG2, SWEET11, SUC2, NPF2.11	(Jin et al., 2024)

*NR, not reported.

In other medicinal plants bearing glandular trichomes, single-cell and spatial omics technologies have similarly advanced our understanding of trichome metabolic compartmentalization and developmental regulation. In a spatial context, the integration of single-nucleus transcriptomics and spatial transcriptomics confirmed, that artemisinin biosynthetic genes are predominantly enriched in secretory cells in *Artemisia annua*, thereby validating the compartmentalized organization of the artemisinin pathway at cellular resolution (Zhang et al., 2025). Analogously, in *Artemisia argyi*, the same methodological framework was applied to systematically resolve the metabolic differences between glandular and non-glandular trichomes and their respective cellular origins. Metabolomic analyses revealed significant enrichment of sesquiterpenoids and other secondary metabolites in glandular trichomes, while single-cell transcriptomics further constructed a leaf cell atlas in which sub-cluster re-analysis precisely defined trichome cell types and their developmental trajectories (Dong et al., 2025).

Collectively, these cases demonstrate that within glandular trichomes, single-cell and spatial omics technologies can transcend the resolution limitations of conventional tissue-level analyses, enabling precise localization of metabolic pathway enzymes, transporters, and regulatory factors to specific cell types. This technological approach provides important methodological implications for further elucidating the biosynthesis of secondary metabolites and their intercellular transport mechanisms within glandular trichomes.

## 5 Transport and trafficking routes

### 5.1 Transmembrane transporter families in the transport network of compartmentalized metabolism

The biosynthesis of plant specialized metabolites frequently exhibits pronounced spatial compartmentalization, as amply demonstrated by the preceding case studies. Nicotine biosynthesis in tobacco exemplifies a canonical “inter-organ” pathway. The MIA biosynthesis in *Catharanthus roseus* operates through “intercellular” compartmentalization. And cannabinoid biosynthesis is predominantly carried out within glandular trichome cells through subcellular partitioning among plastids, the cytoplasm, and extracellular storage cavities. It is thus evident that the accumulation of alkaloids and related specialized metabolites is not solely determined by biosynthetic enzymes. Their dynamic partitioning and intercellular transport within the plant are equally critical for the continuous operation of these pathways. In this context, multiple transmembrane transporter families have been demonstrated or proposed to participate in the transport and regulation of intermediates and final products, including the ATP-binding cassette (ABC), MATE, PUP, and the more recently highlighted NPF families (Shitan et al., 2014a). have systematically reviewed alkaloid transport across multiple levels — inter-organ, intercellular, and subcellular — and provided detailed accounts of the roles of ABC, MATE, and PUP family members in alkaloid efflux, vacuolar sequestration, and uptake-recycling (Shitan et al., 2014a).

Single-cell and spatial omics technologies have introduced a new resolution scale for localizing these transporters to specific cell types. In Catharanthus roseus, single-cell transcriptomics not only revealed the fine-grained division of MIA pathway genes across distinct cell populations, but also identified candidate transporters specifically expressed in IPAP cells, epidermal cells, and idioblasts, which provides cell-resolution evidence in support of the intercellular transport models proposed by classical studies (Sun et al., 2023). Analogous strategies have been applied in other trichome metabolic systems. In tobacco, single-nucleus transcriptomic atlases distinguished LGT and SGT cell clusters, providing a cellular context for interpreting the cell type-specific expression of nicotine-related MATE and PUP transporters. In Artemisia annua (Hildreth et al., 2011; Chen et al., 2024), snRNA-seq combined with spatial transcriptomics has anchored the artemisinin biosynthetic module to secretory cells, further demonstrating the broad applicability of transport network analysis at single-cell resolution (Zhang et al., 2025).

Notably, the discovery of NPF family members has further broadened the scope of specialized metabolite transport research. Originally regarded as primarily involved in nitrate and peptide transport, mounting evidence indicates that NPF members have been recruited for the transmembrane transport of specialized metabolic intermediates (Kanstrup and Nour-Eldin, 2022). In *Catharanthus roseus*, Payne et al. identified CrNPF2.9, a tonoplast-localized transporter that exports the key intermediate strictosidine from the vacuole to the cytoplasm. This transport step is required for the MIA pathway to proceed continuously (Payne et al., 2017). This finding indicates that the vacuole functions not merely as a terminal storage compartment, but may also serve as a dynamic hub for intermediate metabolite flux.

### 5.2 Vesicle-mediated transport & cuticular deposition

Beyond active transport mediated by transmembrane transporters, the glandular trichome secretory system also involves vesicle-mediated transport and cuticular deposition mechanisms, which are particularly prominent in systems with high accumulation of hydrophobic metabolites. In the cannabinoid pathway, the terminal oxidative cyclase THCAS is secreted into the subcuticular storage cavity to complete its catalytic reaction, indicating that the biosynthetic enzyme itself may be transported to the extracellular space via a vesicular route to carry out terminal biosynthesis, with subsequent accumulation within the cavity (Sirikantaramas et al., 2005). The highly active endomembrane system and secretory cavity architecture of glandular trichome cells are likewise considered to constitute an important morphological basis for their function as metabolic cell factories. The integrity of the cuticle directly influences metabolite storage and stability. The tobacco trichome-specific lipid transfer protein *Nicotiana tabacum* Lipid Transfer Protein 1 (NtLTP1) localizes to the secretory structures of glandular trichomes and has been shown to be essential for lipid efflux and secretion formation, suggesting that lipid transfer proteins may act in concert with ABCG-type transporters to participate in cuticular deposition and the efflux of hydrophobic products (Hildreth et al., 2011). The terminal accumulation of trichome metabolites therefore depends not only on transmembrane transport, but also involves vesicle-mediated secretion and cuticular structural remodeling. Together, these processes form a coupled secretory system that relies on multiple mechanisms.

In summary, single-cell and spatial omics technologies have substantially enhanced the capacity to resolve specialized metabolite transport networks. By simultaneously localizing biosynthetic enzyme genes, transporters, and metabolites at cellular resolution, these technologies have transcended the resolution limitations of conventional bulk tissue analyses, enabling the direct validation and integration of inter-organ, intercellular, and subcellular transport routes. They have not only clarified the spatial dissociation between biosynthetic and accumulation sites, but also provided a critical foundation for identifying key transport nodes and constructing spatially resolved metabolic network models. As multi-omics integration strategies continue to advance, single-cell and spatial technologies will sustain the systematic deepening of our mechanistic understanding of metabolite transport in glandular trichomes and other specialized tissues.

## 6 Emerging themes and knowledge gaps

With the rapid advancement of single-cell and spatial omics in studies of plant glandular trichomes, intercellular heterogeneity previously obscured by tissue-averaging effects has been systematically revealed for the first time (Liu et al., 2021). By reconstructing metabolic and regulatory landscapes at single-cell resolution, researchers can now identify functional state differences within trichome cell populations, their spatial organizational patterns, and intercellular signaling interactions (Dong et al., 2025; Zhang et al., 2025). These technologies facilitate the understanding of the spatial architecture of complex secondary metabolic networks, while simultaneously exposing a wealth of unresolved key scientific questions.

At the level of metabolic regulation, transcription factors govern the directionality and flux intensity of metabolism by coordinately regulating multiple metabolism-associated genes (Wang et al., 2021). As secondary metabolic pathways involve multi-enzyme cascade reactions and are readily influenced by environmental perturbations (Pichersky and Lewinsohn, 2011; Schmidt‐Dannert and Lopez‐Gallego, 2016), single-gene modifications alone frequently fail to yield optimal outcomes. Single-cell data reveal that within specific trichome subtypes, transcription factors regulating a given metabolic pathway often exhibit co-expression patterns with the transporters responsible for exporting the corresponding metabolites, forming precise “modular” regulatory units (Huang et al., 2023). Upon pathway activation, the associated transporter genes are concurrently induced, maintaining efficient and directional metabolic flux while preventing intermediate accumulation and potential cytotoxic buildup (Tohge et al., 2005). This phenomenon indicates that our understanding of the transcriptional coordination mechanisms underlying trichome metabolism remains incomplete.

Traditional studies have shown that phytohormones such as jasmonic acid and abscisic acid can induce secondary metabolism (Ferrandino and Lovisolo, 2014; Zhang et al., 2023), but glandular trichomes are often treated as a unified, homogeneous response unit. However, single-cell analyses reveal that distinct trichome cell subtypes differ markedly in their sensitivity to hormonal signals (Boughton et al., 2005; Maes and Goossens, 2010). Single-cell technologies can not only precisely identify which specific subtypes respond first to hormonal stimulation, but also resolve how hormones precisely remodel the transcriptional networks of target cell populations (Zhang et al., 2023). This helps to elucidate the mechanisms underlying hormone-induced activation of defense metabolic pathways at a higher resolution. This raises a critical question: is hormonal regulation in glandular trichomes spatially partitioned? It also remains unclear whether distinct cell subtypes serve specific roles in decoding hormonal signals. These knowledge gaps warrant further investigation.

Although, the advancement of single-cell and spatial omics technologies are emerging other technical challenges still hinders the applications of these technologies in GT research. One of the major hinderance is the lower abundance of GT tissues for analysis. In addition, GTs are highly fragile to handle which results in mechanical disruption during tissue processing this can rupture secretory cavities and lead to loss or redistribution of metabolites. This can affect the accuracy of spatial and structural characterization of specialized metabolic pathways. Moreover, the single cell OMICS requires isolation of protoplast which can trigger the stress-responsive cellular/transcriptional mechanism which might pose noise for the native gene expression and lead to confounding effects. Even though the snRNA-seq by-passes protoplast isolation and primarily depicts the nuclear transcripts. This could underestimate the expression of transcripts present in the cytoplasm, plastids and mitochondria. Therefore, the above-mentioned technical limitations should be carefully considered during the interpretation of cell-type-specific metabolic pathways and transcript regulations/expression.

## 7 Future perspectives

In the biosynthesis and transport of secondary metabolites in plant cells, transcriptional regulation represents only one of many contributing factors. Protein-level dynamics and metabolite flux are equally integral in shaping cellular function (De la Cruz-Velueta et al., 2024). The trajectory of future research will therefore inevitably shift from single-dimensional analyses toward multidimensional integration. The concurrent application of single-cell transcriptomics (gene expression), single-cell assay for transposase-accessible chromatin (scATAC-seq) (chromatin accessibility, reflecting regulatory state), and single-cell or spatial metabolomics (metabolite distribution) to the same cell type holds the promise of directly constructing causal chains from regulatory switches through expression changes to metabolic function (Jang et al., 2025; Luo et al., 2025), substantially enhancing our capacity to dissect secondary metabolic mechanisms. Although examples integrating high-throughput spatial transcriptomics, scRNA-seq, scATAC-seq, and MSI in plant research remain limited (Yu et al., 2023), this strategy has already demonstrated considerable success in animal developmental studies (Fernandes et al., 2021; Chen et al., 2023) and hold substantial potential for application in plant glandular trichomes.

For research on secondary metabolism in glandular trichomes, the ultimate goal of integrative multi-omics is to decode the “design blueprint” of native trichomes. This could enable precise reprogramming of pathways in model microorganisms or crop plants to build cell factories that efficiently produce high-value natural products (Tissier, 2012). It may also support the engineering of artificial trichome-like structures with entirely new metabolic functions. Realizing this vision is critically dependent on a deep mechanistic understanding of metabolite biosynthesis, intercellular transport, and subcellular storage within trichome cells, with transporter functional characterization representing one of the most pivotal bottlenecks (Wen et al., 2025). With the rapid advancement of artificial intelligence, large-scale models trained on vast scRNA-seq datasets can predict the likely substrates of transporters based on their co-expression patterns with metabolic pathway genes across thousands of individual cells, thereby substantially accelerating the screening and functional validation of candidate transporters (Li et al., 2024; Xiao et al., 2024).

Concurrently, single-cell resolution atlases enable researchers to identify highly specific promoters that are activated exclusively in particular trichome cell types (Conneely et al., 2024). By harnessing these promoters, metabolic engineering constructs can be precisely targeted for expression in cells of interest. such as trichome head cells. This can avoid interference with whole-plant growth and enable cell-type-specific precision metabolic engineering, thereby improving the biosynthetic efficiency of target compounds.

In summary, key genes, regulatory elements, and specific cell types identified by single-cell omics will provide directly actionable targets for molecular marker-assisted breeding and genetic engineering. These efforts can increase the content of medicinal compounds, improve flavor quality, or enhance industrial feedstock yields, thereby accelerating innovation across the agricultural and biotechnology industries (Schwekendiek et al., 2007; Schilmiller et al., 2008; Glas et al., 2012; Tissier, 2012). Broadly speaking, the field is advancing from descriptive discovery toward mechanistic elucidation, and ultimately toward engineering application, with the overarching goal of establishing sustainable and scalable new paradigms for natural product biomanufacturing.

## 8 Conclusion

Single-cell and spatial omics technologies have provided unprecedented insights into alkaloid and terpenoid biosynthetic pathways in plant glandular trichomes. The application of these technologies has transcended the limitations of conventional omics research, revealing the critical roles of distinct cell types and their interactions in trichome metabolic processes. By constructing metabolic and regulatory maps at cellular resolution, researchers can precisely localize key biosynthetic enzymes, transporters, and storage structures, enabling a systematic description of the complete “biosynthesis–transport–storage” continuum of secondary metabolites. This paradigm shift from “tissue-level ambiguity” to “cell-level clarity” provides a more reliable empirical foundation and theoretical framework for both metabolic pathway elucidation and engineering applications. Furthermore, the convergence of single-cell and spatial omics creates new opportunities for synthetic biology and breeding technologies. By identifying cell type-specific promoters, key genes, and regulatory elements, researchers can carry out highly precise molecular design and trait improvement. This enables targeted control of specialized metabolic traits in crops and opens new avenues for agriculture, medicine, and the natural products industry.

Overall, these technologies have not only advanced a more refined mechanistic understanding of secondary metabolism in glandular trichomes but have also laid a solid foundation for the construction of efficient and controllable biomanufacturing systems for plant natural products. As technological progress continues, single-cell and spatial omics will exert an increasingly profound influence across biology, synthetic biology, and agricultural science, offering novel conceptual frameworks and engineering solutions to address global challenges in agriculture and medicine.
